# Pharmacokinetics in Wistar Rats of 5-[(4-Carboxybutanoyl)Amino]-2-Hydroxybenzoic Acid: A Novel Synthetic Derivative of 5-Aminosalicylic Acid (5-ASA) with Possible Anti-Inflammatory Activity

**DOI:** 10.1371/journal.pone.0159889

**Published:** 2016-07-25

**Authors:** Aurelio Romero-Castro, Mara Gutiérrez-Sánchez, José Correa-Basurto, Martha Cecilia Rosales Hernández, Itzia Irene Padilla Martínez, Jessica Elena Mendieta-Wejebe

**Affiliations:** 1 Laboratorio de Biofísica y Biocatálisis, Sección de Estudios de Posgrado e Investigación, Escuela Superior de Medicina, Instituto Politécnico Nacional, Plan de San Luis y Salvador Díaz Mirón s/n, Casco de Santo Tomás, Ciudad de México 11340, México; 2 Laboratorio de Modelado Molecular y Bioinformática, Sección de Estudios de Posgrado e Investigación, Escuela Superior de Medicina, Instituto Politécnico Nacional, Plan de San Luis y Salvador Díaz Mirón s/n, Casco de Santo Tomás, Ciudad de México 11340, México; 3 Laboratorio de Química Supramolecular y Orgánica, Departamento de Ciencias Básicas, Unidad Profesional Interdisciplinaria de Biotecnología, Instituto Politécnico Nacional, Av. Acueducto s/n, Barrio La Laguna Ticomán, Ciudad de México 07340, México; Hungarian Academy of Sciences, HUNGARY

## Abstract

5-[(4-carboxybutanoyl)amino]-2-hydroxybenzoic acid (***C2***) is a novel synthetic derivative of 5-aminosalicylic acid (5-ASA), which is currently being evaluated *ex vivo* as an anti-inflammatory agent and has shown satisfactory results. This study aimed to obtain the pharmacokinetic profiles, tissue distribution and plasma protein binding of ***C2*** in Wistar Rats. Additionally, an HPLC method was developed and validated to quantify ***C2*** in rat plasma. The pharmacokinetic profiles of intragastric, intravenous and intraperitoneal administration routes at singles doses of 100, 50, and 100 mg/kg, respectively, were studied in Wistar rats. The elimination half-life of intravenously administered ***C2*** was approximately 33 min. The maximum plasma level of ***C2*** was reached approximately 24 min after intragastric administration, with a C_max_ value of 2.5 g/mL and an AUC_tot_ value of 157 μg min^-1^/mL; the oral bioavailability was approximately 13%. Following a single intragastric or oral dose (100 mg/kg), ***C2*** was distributed and detected in all examined tissues (including the brain and colon). The results showed that ***C2*** accumulates over time. The plasma protein binding results indicated that the unbound fraction of ***C2*** at concentrations of 1 to 20 μg/mL ranged from 89.8% to 92.5%, meaning that this fraction of ***C2*** is available to cross tissues. Finally, the blood-plasma partitioning (BP ratio) of ***C2*** in rat plasma was 0.71 and 0.6 at concentrations of 5 and 10 μg/mL, respectively, which indicates that ***C2*** is free in the plasmatic phase and not inside blood cells. The results of this study suggest that a fraction of the administered ***C2*** dose is absorbed in the stomach, and the fraction that is not absorbed reaches the small intestine and colon. This distribution constitutes the main advantage of ***C2*** compared with 5-ASA for the treatment of ulcerative colitis (UC) and Crohn's disease (CD).

## Introduction

Inflammatory bowel disease (IBD) is the medical term used to describe chronic inflammatory diseases of the gastrointestinal tract (GI) that are characterized by a wide range of signs and symptoms, such as diarrhea, abscesses, fistulas, abdominal pain and stenosis. These symptoms significantly affect the quality of life of affected patients [1, 2]. Although the symptoms of Crohn’s disease (CD) and ulcerative colitis (UC) are quite similar, they affect different areas in the GI are different. Specifically, CD most commonly affects the end of the small bowel (the ileum) and the beginning of the colon, but it may affect any part of the GI tract, from the mouth to the anus. Conversely, UC inflammation typically arises in the distal colon and extends in a proximal direction [3–6]. Both CD and UC are characterized by periods of active intestinal inflammation that may require hospitalization [1, 2, 5, 7, 8].

The main goal of pharmacological IBD treatment is the reduction of inflammatory process during relapses [9–13]. 5-aminosalicylates are non-steroidal anti-inflammatory drugs (NSAIDS) characterized by their analgesic, antipyretic, and anti-inflammatory effects. These drugs are used in conventional therapies to decrease the exacerbated immune responses in CD or UC in patients. Sulfasalazine, mesalazine, olsalazine, and balsalazide are 5-aminosalicylates that are used to treat UC, but their use in patients with CD remains controversial [13–17]. These compounds act locally on the colonic mucosa, but unfortunately, they are associated with side effects, such as diarrhea, nausea, vomiting, headache, abdominal pain, fatigue, weaknesses, hepatic abnormalities, arthralgia and myalgia. Specifically, the side effects of mesalazine (5-aminosalicylic acid) have been related to the sulfapyridine component [18, 19].

Uncoated 5-aminosalicylic (5-ASA) is absorbed in the small intestine when orally administered. Therefore, it cannot reach the colon mucosa in its unchanged form [20–24], and 5-ASA is consequently currently administered in oral formulations of delayed or controlled release and as a prodrug (sulfasalazine, olsalazine, balsalazide) [25–31].

**Fig 1:**
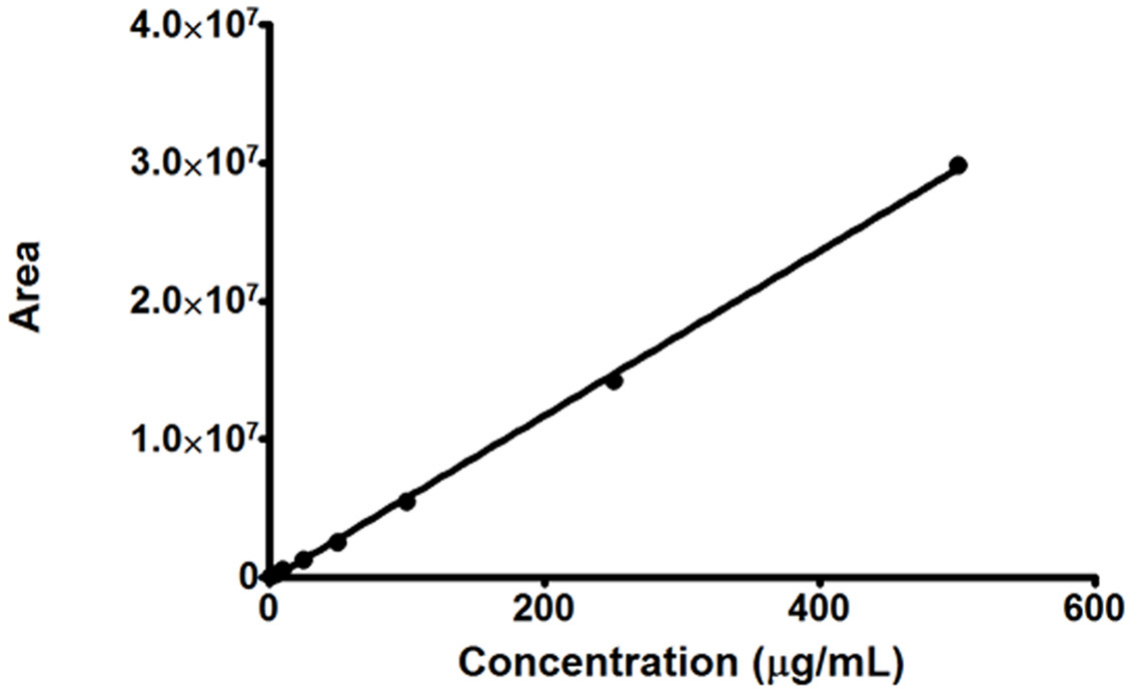
Calibration curve of *C2*.

Several research groups have designed new anti-inflammatory molecules that inhibit myeloperoxidase (MPO) [32]. Our group designed [33] and synthesized 5-[(4-carboxybutanoyl)amino]-2-hydroxybenzoic acid (***C2***) (Fig 1), which has shown satisfactory results in pharmacologic and toxicological studies [34–36]. Specifically, ***C2*** inhibited the catalytic activity of MPO in a model of inflammation in the mouse ear [34] and was nontoxic in CD1 mice and Wistar rats (LD_50_ > 2000 mg/kg) [37]. In this work, the pharmacokinetic profiles (intragastric, intravenous and intraperitoneal) and distribution of ***C2*** in rats were studied to assess the ability of ***C2*** to reach the rat colon, which is a necessary condition for its local pharmacologic affect. To quantify ***C2*** in the plasma, organs and tissues, a bio-analytical method was developed and validated using High Performance Liquid Chromatography (RP-HPLC).

## Material and Methods

### Chemicals and Standards

Acetonitrile, methanol and water (HPLC grade) were purchased from Tecsiquim (Mexico). Acetic acid (analytical grade), polysorbate 80 (tween) and propylene glycol were purchased from Sigma–Aldrich (St. Louis, MO, USA). Heparin (1000 UI/mL) and 0.9% sodium chloride were purchased from PISA (Mexico). The anesthetics ketamine (CLORKETAM^®^) and xylazine PROCIN^®^) were intended for veterinary use and acquired from Vétokinol (Lure Cedex, France) and PISA Agropecuaria, S.A. de C.V. (Hidalgo, Mexico), respectively.

A batch of ***C2*** was synthesized at the Laboratorio de Investigación en Química Orgánica y Supramolecular de la Unidad Profesional Interdisciplinaria de Biotecnología; the molecular structure of ***C2*** was validated using infrared spectroscopy, ^1^H nuclear magnetic resonance spectroscopy and ^13^C and mass spectrometry; these analyses were conducted by the Centro de Nanociencias y Micro y Nanotecnología-IPN. The purity of ***C2*** was assessed by HPLC. This batch of ***C2*** was used as the reference standard due to its high purity (98.7%).

### *C2* Formulation for Administration

The intravenous formulation was a translucent white solution prepared by dissolving 100 mg of ***C2*** in 10 mL of a mixture consisting of propylene glycol-polysorbate 80-sodium chloride (0.9%) at a ratio of 20:5:75, (v/v/v), respectively. The solution was vortexed for 3 min and sterilized by filtration before being filtered with a 0.22 μm nylon syringe filter; the final concentration was approximately 10 mg/mL.

### Ethics Statement

The animal procedures were conducted in accordance with the Mexican Official Standard NOM-062-ZOO-1999, Technical Specifications for Production, Care and Use of Laboratory Animals [38]. The animal protocol was approved by the Research Committee for the Care and Use of Laboratory Animals (CICUAL) of the Escuela Superior de Medicina-IPN (Approval number: ESM.CICUAL-02/27-07-2015; S1 File).

In this study, the experiments were finished by the experimental endpoint. However, all animals were monitored for early indicators of a human endpoint [39]. After the collection of samples, the animals were sacrificed with an intraperitoneal dose of 72 mg/kg sodium pentobarbital.

### Animals

Male Wistar rats were obtained from the Bioterio of the Escuela Superior de Medicina, Instituto Politécnico Nacional. For all experiments, the animals were placed in polycarbonate cages during the acclimation (one week) period and the experiments. The diet consisted of Rat Chow 5012 (Purina) and water *ad libitum*. For the pharmacokinetic studies, the animals were randomly divided into three groups (n = 6, body weight 300 ± 20 g). The rats were fasted for 8 h before dosing but allowed free access to water.

### Rat Plasma Collection for Validation

Plasma was obtained from rats using a cardiac puncture protocol. Briefly, the animals were anaesthetized with sodium pentobarbital, and cardiac puncture was rapidly performed; the blood samples were then collected into heparinized tubes. The plasma was separated by centrifugation at 8,000 rpm and 4°C for 15 min and stored at– 80°C until use.

### Instrumentation and Analytical Conditions

The levels of ***C2*** were quantified using an Agilent 1260 Infinity Series liquid chromatograph (Agilent Technologies, Palo Alto, CA, USA) equipped with a quaternary pump delivery system (G1311B), robotic autosampler (G1316A), column thermostat (G1316A), and multi-wavelength UV detector (G1315C); the results were analyzed using OpenLab CDS EZChrom. A Zorbax SB-C18 column (5 μm, 4.6 x 150 mm, Agilent Technologies, Palo Alto, CA, USA) was used for separation, and UV detection was carried out at 254 nm. The column temperature was maintained at 45°C, and the injection volume was 10 μL. The mobile phase consisted of a mixture of (A) 0.2% acetic acid in water (v/v), pH 3.0 and (B) acetonitrile at a ratio of 20% A and 80% B and flow rate of 0.6 mL/min. The total analysis time of each sample was 6.0 min using an isocratic elution, and equilibrating between each injection was not required.

### Preparation of Calibration Standards and Quality Control (QC) Samples

Six stock solutions (1.0 mg/mL) were prepared by dissolving the appropriate weight of the compound in a mixture of methanol (50:50, v/v). These samples were stored at 2–4°C and were stable for one month. Calibration standards were prepared daily by diluting stock solutions. Briefly, 100 μL of blank plasma was spiked with an appropriate volume of stock solution, and the samples were then vortexed for 5 min. Next, appropriate volumes of acetonitrile-methanol (50:50) were added until each sample reached a final volume of 1 mL; these samples were centrifuged at 8,000 rpm for 10 min, and the supernatant was subsequently filtered using a nylon syringe filter with a 0.45 μm pore size. A volume of 10 μL was injected into the HPLC system. The standard solutions were prepared in triplicate for the eight calibration points, and the final concentrations obtained were 1, 2.5, 5, 10, 25, 50, 100, 250 and 500 μg/mL. Finally, calibration curves were constructed in triplicate by plotting known concentrations of the standard versus the detector response area.

A separate set of stock solutions was prepared for the QC samples for use in the method validation to assess accuracy and precision. All QC samples were prepared over three different concentration ranges in triplicate. The calibration and QC samples were prepared on different days. Furthermore, the QC samples were prepared independently using plasma from untreated rats.

### Method Validation

The method was validated according to the FDA Industry Guidance for the Bioanalytical Method Validation and ICH Harmonised Tripartite Guideline for the Validation of Analytical Procedures [40, 41]. The following parameters were evaluated: linearity, intra- and inter-day accuracy and precision, recovery, the lower limit of quantification (LLOQ), the limit of detection (LOD), selectivity, and stability.

#### Linearity

Linearity was determined as follows: A nine-point calibration curve was constructed by analyzing the standards in the concentration range mentioned above. Linearity was measured for the selected concentration range by fitting the data to a linear regression model and assessing the coefficient of determination (R^2^ ≥ 0. 980) [40, 41].

#### Intra- and Inter-Day Accuracy and Precision

These parameters were determined by analyzing different QC samples (n = 5) at high, medium, and low concentrations of ***C2*** (1, 25, and 100 μg/mL) on five consecutive days. The accuracy and precision are expressed in terms of the relative error (RE) and relative standard deviation (RSD), respectively. The intra- and inter-day precision should not exceed 15%, and the accuracy should be within ± 15% for the QC samples [40, 41].

#### Recovery

The extraction recovery of ***C2*** in rat plasma was assessed by comparing the mean peak areas of the processed QC samples with those of the corresponding the blank plasma matrix samples spiked with standard solutions, which were similarly prepared and had the same final concentration, except water replaced the blank plasma. The recovery of ***C2*** was assessed at 1, 25, and 100 μg/mL [40, 41].

#### Lower Limit of Quantification (LLOQ) and Limit of Detection (LOD)

The LLOQ was determined from the last point of the calibration curve, and the average recovery value was ± 20% of the nominal value with a coefficient of variation (C.V.) ≤ 20%. The LOD was calculated according to the following equation: LOD = 3.3 σ/S, where σ represents the standard deviation of the intercepts of the regression lines, and S represents the mean of the slopes of the calibration curves of the analyte (ICH, 2005) [41].

#### Selectivity and Specificity

Selectivity is the ability to accurately and specifically measure the analyte in the presence of components that may be expected to be present in the sample matrix. This parameter was investigated by analyzing blank plasma samples from six rats at the LLOQ concentration, which were then compared with the corresponding plasma samples spiked with ***C2*** [40, 41].

In addition, we analyzed the potential interference of heparin, ketamine and xylazine, which were used in this pharmacokinetic study. Hence, the specificity was determined by assessing the peak identity and purity of ***C2***. A peak purity angle that is less than the peak threshold angle is an indication of the spectral homogeneity or purity of ***C2***.

#### Stability

The stability of ***C2*** in plasma was evaluated using the QC samples at two concentrations by triplicate (high = 100 μg/mL, medium = 25 μg/mL and low = 1 μg/mL); these samples were stored at 25°C for 12, 24 and 36 h (short-term stability) and subjected to three freeze-thaw cycles (–20°C to 25°C). The post-preparative storage was evaluated by analyzing the ready-to-inject samples that had been refrigerated 4°C for 12, 24 and 36 h. The concentration of ***C2*** after each storage period was related to the initial concentration of freshly prepared and immediately processed samples [40, 41].

### Pharmacokinetic Studies

Prior to the studies (on the day of ***C2*** administration), the Wistar rats (n = 6) were cannulated. For intravenous (i.v.) treatment, a 24 G catheter was placed in the right lateral tail vein (for blood collection), and another catheter was placed in the left (for ***C2*** administration) lateral tail vein of the rat as follows: the animal was placed inside a trap (special acrylic stocks for rats), the rat tail was exposed, and the distal region of the tail was disinfected with 70% ethanol; the tail veins were then dilated using a heating pad (approximately 45°C). An assistant held the tail of the rat while the left lateral vein was located, and a 24 G catheter was then firmly inserted into the posterior third of the caudal vein of the animal, where it was fixed with adhesive tape (for intragastric and intraperitoneal studies, only one catheter was placed on the right for blood collection).

For the intravenous study, a single dose of 50 mg/kg ***C2*** was administered through the 24 G catheter when the rat was inside the trap. For the intragastric (i.g) pharmacokinetic study, a second group of rats (n = 6) received a single dose of 100 mg/kg using a 20 G gavage needle (animals were provided with a standard diet 4 h after dosing). For the intraperitoneal (i.p.) treatment, a third group of rats (n = 6) was injected with 75 mg/kg ***C2***. In all pharmacokinetic studies, approximately 250 μL of blood was collected in heparinized tubes from each rat via the catheter in the right lateral tail vein prior to dosing and 5, 10, 20, 30, 45, 60, 90, 120, 180, 240, 300, 360, 540, 720 and 1440 min after dosing. These blood samples were supplemented with an equal volume of 0.9% sodium chloride solution. Plasma (100 μL) was harvested by centrifuging the blood samples at 8,000 rpm for 15 min, and the plasma was then immediately processed for analysis.

#### Sample Preparation

The samples were prepared as follows: briefly, 450 μL of acetonitrile was added to 100 μL of rat plasma, and the mixture was vortexed for 5 min. Subsequently, 450 μL of methanol was added to the sample, which was then vortexed again and centrifuged to precipitate proteins. Subsequently, 10 μL of the supernatant was introduced into the HPLC system. The plasma concentrations of ***C2*** were determined using an RP-HPLC with UV detection method that had previously been validated for pharmacokinetic studies and the complementary assays performed in this work.

#### Data Analysis

The plasma concentration–time data obtained after the i.v., i.g., and i.p. administration of ***C2*** were subjected to a non-compartmental analysis using statistical moment theory. The pharmacokinetic parameters of ***C2*** were calculated using the Kinetica 5.0 software (Adept Scientific Ltd), including the maximum plasma concentration (C_max_), time to reach C_max_ (T_max_), elimination half-life (t_1/2_), area under the plasma concentration-time curve from time zero to the last measurable concentration (AUC_0-t_), area under the plasma concentration-time curve from time zero to infinity (AUC_tot_), mean residence time (MRT), clearance (CL), and apparent volume of distribution (Vd).

#### Tissue Distribution Study

Another group of fifteen male Wistar rats was orally administered a single 75 mg/kg dose of ***C2***. The animals were sacrificed 10, 30, 90, 180 and 360 min after dosing (n = 3 per treatment time). The whole brain, heart, liver, spleen, lung, kidney, stomach, small intestine, colon, testicles and muscle were rapidly dissected, harvested and thoroughly rinsed in ice-cold saline to eliminate blood and other content. All tissues and organs were weighed on an analytical balance and immediately processed for analysis. Each tissue sample was homogenized with saline solution at a 1:3 (wt/v) ratio. The preparation process for analysis was the same as that described above for plasma [42].

#### Blood-Plasma Partitioning (BP Ratio)

Whole blood was spiked with different amounts of ***C2*** to obtain final concentrations of 5 and 20 μg/mL, and these samples were then incubated at 37°C for 4 h (samples were prepared by triplicate). The plasma was separated from blood samples, and the concentration of ***C2*** was determined based on a standard curve prepared with blank plasma using the RP-HPLC method. The blood/plasma concentration ratio (BP) was determined by dividing 5 and 20 μg/mL by the concentration found in plasma sample. The concentration of ***C2*** in blood cells was assumed to be equal to its unbound concentration in the plasma [43].

#### Plasma Protein Binding Assay

To assess the plasma protein binding of ***C2*** using the ultrafiltration method, 600 μL samples of freshly obtained plasma from untreated rats (blank) were spiked with different amounts of ***C2*** (10 mg/mL) to give final concentrations of 1, 5, 20 and 30 μg/mL. The resulting samples were incubated for 4 h at 37°C. After incubation, a 100 μL aliquot was removed to analyze the total concentration, and the remaining 500 μL was transferred to a 10 kD cut-off ultrafiltration device (Millipore Corporation, USA), which was centrifuged at 2,000 g for 2 h at 37°C. A 100 μL aliquot of centrifuged plasma was analyzed for the free drug concentration using the RP-HPLC method. The amount of the test compound in the ultrafiltrate was determined by interpolating the main areas of the samples on standard curves containing known amounts of ***C2***. The percentage of protein binding was calculated using the following formula: Protein binding ratio (%) = [(1 –(drug ultrafiltrate)) / (drug total)] x 100. Additionally, the use of protein-free plasma instead of plasma indicated that ***C2*** minimally bound to the ultrafiltration device [42, 43].

## Results

### Method Validation

The chromatographic conditions, especially the composition of the mobile phase, were optimized throughout several trials to achieve a good resolution and symmetric peak shapes for ***C2***. Modifications, such as phosphoric acid and acetic acid alone or in combination at different concentrations, were tested. After testing several conditions, an isocratic elution of the mobile phase consisting of acetonitrile and 0.2% acetic acid (80:20) using a Zorbax SB C-18 column (150 mm × 4.6 mm, 5 μm) was found to yield a good peak shape and a suitable retention time of 10.2 min.

***C2*** was extracted to avoid plasma interference and achieve good and consistent recovery. Conventional methodologies for protein precipitation were tested; protein-precipitating agents, such as acids, methanol, chloroform, ethyl acetate, and dichloromethane alone or in combination at different proportions, were investigated to efficiently extract ***C2***. We developed a two-step liquid-liquid extraction procedure from plasma using 450 μL of acetonitrile and 450 μL of methanol versus 100 μL of plasma, which excellently recovered ***C2*** (above 90%): a clean chromatogram for a blank plasma sample and a high recovery of ***C2*** from plasma were obtained.

#### Linearity

All plasma calibration curves for ***C2*** were linear over the concentration range of 1 to 500 μg/mL (Fig 1) with a correlation coefficient (R) greater than 0.99. A typical linear regression equation for the calibration curve was y = 59390x + 256011 (R^2^ = 0.9990), where y represents the area of the peak of ***C2*** and x represents the concentration of ***C2*** in the sample.

The values of the LLOQ and the LOD were found at 1 μg/mL for both parameters with a C.V. of 5.9%.

#### Intra- and Inter-Day Accuracy and Precision

The intra- and inter-day accuracy and precision parameters at 1, 25, and 100 μg/mL of ***C2*** are shown in Table 1. The intra-day precisions varied from 2.4% to 5.4, and the accuracies varied from 99.2% to 95.6%. The inter-day precisions varied from 0.7% to 4.2%, and accuracies varied from 86.5% to 96.2%. Thus, the intra-assay and inter-assay accuracy and precision were found to be acceptable and satisfactory for the ***C2*** analysis, in support of further pharmacokinetic studies. The data demonstrated good accuracy and reproducibility, which indicated the applicability of the method to pharmacokinetic studies.

**Table 1:** Intra-day and inter-day (n = 5) accuracy and precision data of the HPLC method for the quantification of C2 in rat plasma.

Nominal concentration (μg/mL)	Measured concentration (μg/mL); mean ± SD	Accuracy (%)	Precision (C.V., %)
**Intra-day**			
1	0.89 ± 0.05	89.2	5.4
25	22.3 ± 0.6	89.2	2.6
100	95.6 ± 2.3	95.6	2.4
**Inter-day**			
1	0.87 ± 0.04	86.5	4.2
25	22.3 ± 0.1	89.1	0.7
100	96.2 ± 1.5	96.2	1.6

#### Recovery

The absolute recoveries of ***C2*** determined at three concentrations, 1, 25 and 100 μg/mL, were 96.0%, 105.7% and 108.9%, respectively. The absolute percent recovery values of ***C2*** are summarized in Table 2.

**Table 2:** Recovery of *C2* from rat plasma in the HPLC method (n = 3).

Nominal concentration (μg/mL)	Recovery (%): mean ± SD	C.V. (%)
1	87.9 ± 3.9	4.4
25	89.6 ± 4.2	4.7
100	95.2 ± 2.1	2.2

#### Selectivity

**Fig 2:**
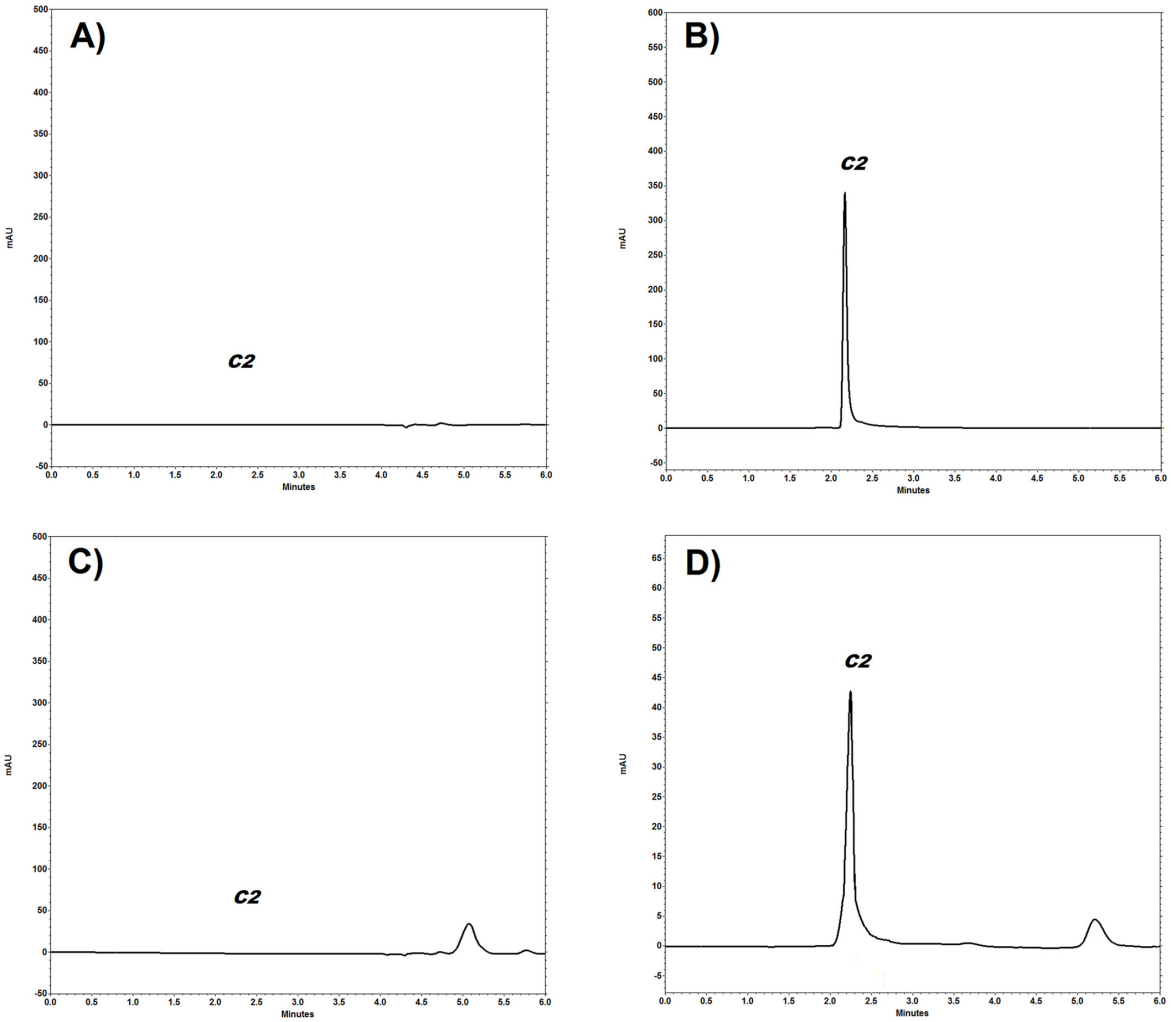
(A) Biological matrix (plasma) chromatogram. (B) Chromatogram of 1 μg/mL *C2* standard. (C) Chromatogram of biological matrix spiked with ketamine, xylazine and heparin (D) Chromatogram of an *in vivo* plasma sample from a rat that had been administered *C2*.

***C2*** was successfully extracted from spiked rat plasma and chromatographically resolved using the previously described sample preparation procedures and chromatographic conditions. Typical chromatograms obtained after the analysis of the ***C2*** of blank and spiked rat plasma samples are shown in Fig 2A and 2B, whereas Fig 2D shows a chromatogram of an *in vivo* plasma sample from a rat that had been administered ***C2***. The retention time of ***C2*** was approximately 2.2 min. The chromatograms in the blank rat matrix showed that no endogenous compounds interfered with the retention times of ***C2***, which indicates that the assay was selective. In addition, under the described chromatographic conditions, none of the tested drugs (ketamine, xylazine and heparin) that are frequently used in experimental protocols of pharmacokinetic studies were found to interfere with the retention time of the chromatographic peak of ***C2*** Fig 2C.

#### Stability

The stability of ***C2*** was investigated under a variety of conditions at four concentrations (1, 2.5, 25 and 100 μg/mL), which were assessed in triplicate, and the results are summarized in Table 3 (stability sample results should be within 15% of nominal concentrations). The data showed that ***C2*** was stable in rat plasma stored at 4°C and 25°C for 36 h, and it was also stable before the three freeze–thaw cycles of the quality control samples.

**Table 3:** Thermal stability of *C2* in rat plasma deproteinized samples (n = 3).

**Condition 1. Room Temperature (25°C)**						
**Time**	**12 h**		**24 h**		**36 h**	
**Concentration (μg/mL)**	**Difference respect the nominal concentration (%)**	**C.V. (%)**	**Difference respect the nominal concentration (%)**	**C.V. (%)**	**Difference respect the nominal concentration (%)**	**C.V. (%)**
1	1.4	5.7	2.1	4.3	5.8	6.8
2.5	1.0	2.2	2.3	3.6	2.6	2.9
25	0.3	2.7	2.9	2.5	3.7	3.5
100	0.3	1.8	0.7	1.2	1.7	3.3
**Condition 2. Refrigeration (4°C)**						
**Time**	**12 h**		**24 h**		**36 h**	
**Concentration (μg/mL)**	**Difference respect the nominal concentration (%)**	**C.V. (%)**	**Difference respect the nominal concentration (%)**	**C.V. (%)**	**Difference respect the nominal concentration (%)**	**C.V. (%)**
1	0.8	5.7	2.2	5.0	5.3	5.7
2.5	1.5	2.2	2.6	4.3	2.9	1.9
25	0.1	2.6	3.1	3.1	4.3	2.8
100	0.5	2.2	0.4	2.0	0.6	2.0
**Condition 3. Three freeze–thaw cycles (From—20 to 25°C)**						
**Time**	**cycle 1 (12 h)**		**Cycle 2 (24 h)**		**Cycle 3 (36 h)**	
**Concentration (μg/mL)**	**Difference respect the nominal concentration (%)**	**C.V. (%)**	**Difference respect the nominal concentration (%)**	**C.V. (%)**	**Difference respect the nominal concentration (%)**	**C.V. (%)**
1	1.8	4.7	2.7	4.9	6.0	1.5
2.5	1.8	2.6	2.9	2.0	5.1	1.4
25	2.0	2.1	1.7	3.0	1.7	1.9
100	0.5	1.95	0.5	2.0	0.6	1.9

### Pharmacokinetic study

**Fig 3:**
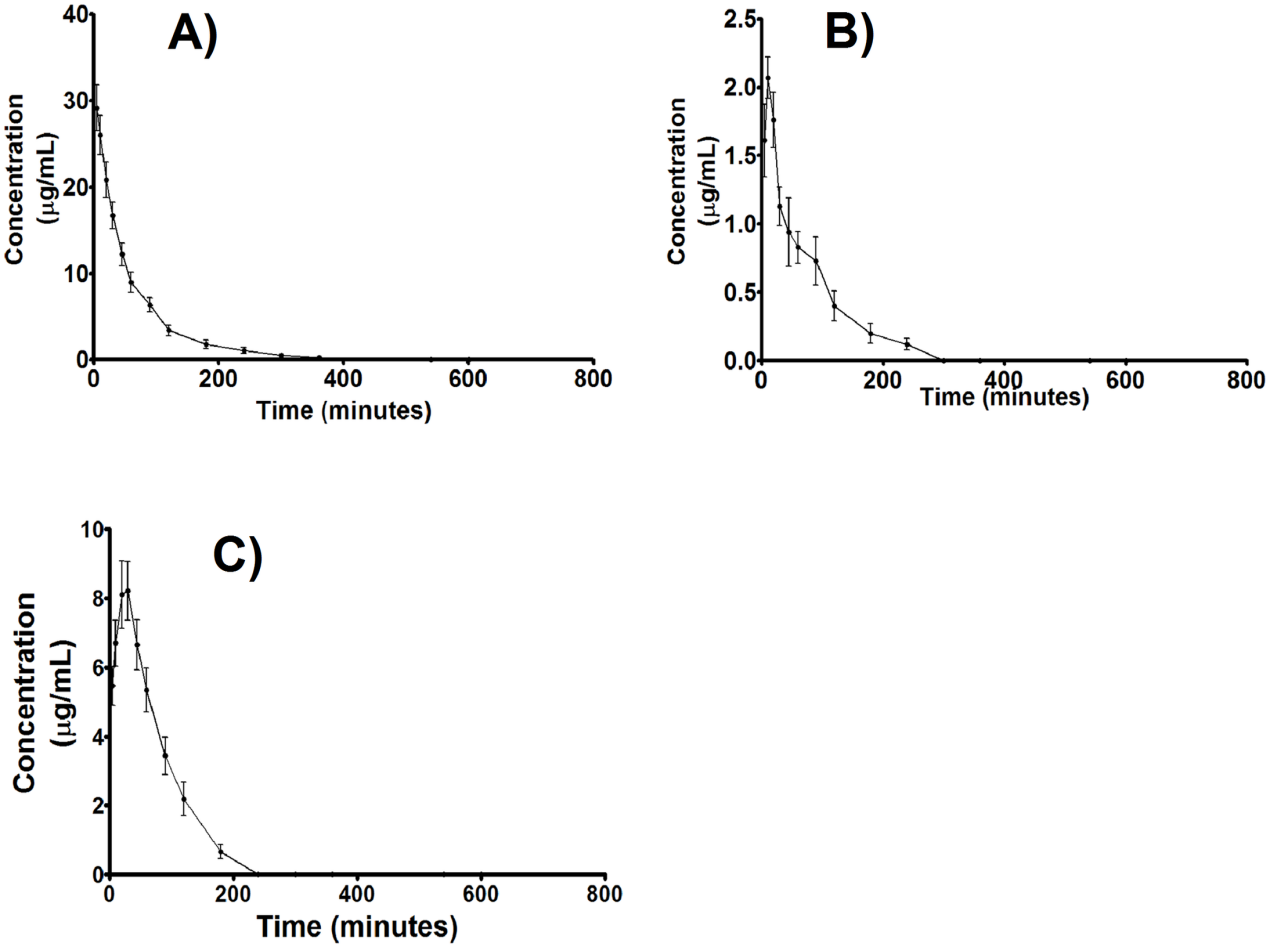
Mean (± SD) of the plasma concentration-time curves of *C2* in rats following (A) intravenous, (B) oral, and (C) intraperitoneal administration (n = 6) using a non-compartmental model.

After the intravenous administration of 50 mg/kg of ***C2***, the plasma concentration declined rapidly (Fig 3A). The MRT value for ***C2*** was 57 ± 11 min, the t_1/2_ was approximately 33 ± 5 min, and the Vd was 2.0 ± 0.2 L/kg. After oral dosing (100 mg/kg), ***C2*** was absorbed, and the maximum plasma levels reached a peak at 24 min (Fig 3B), with a C_max_ value of 2.5 ± 0.3 μg/mL and a t_1/2_ of approximately 27 ± 11 min. The absolute oral bioavailability of ***C2*** was only 13%. The values of all pharmacokinetic parameters for each route of administration are shown in Table 4. As shown in Fig 3, ***C2*** was poorly absorbed at the tested dose (100 mg/kg), reaching maximum plasma levels within 2.5 min and 8.7 min of i.g. (Fig 3B) and i.p. (Fig 3C) administration, respectively. As a consequence, the C_max_ and AUC_tot_ were higher for the i.v. route than i.p. and i.g. routes at the lower dose (Table 4).

**Table 4:** Plasma pharmacokinetic parameters of *C2* determined by a non-compartmental model analysis after a single intravenous, oral, and intraperitoneal administration to Wistar rats at a doses of 50, 75 and 100 mg/kg. Values are presented the as the mean ± SD.

Parameters	Units	Route of administration		
		i.g.	i.p.	i.v.
Ka	h^-1^	-	1.2 ± 0.2	-
C_max_	μg/mL	2.5 ± 0.3	8.7 ± 0.4	-
T_max_	min	24 ± 4	25 ± 2	5 ± 0
Cp^0^	μg/mL	-	-	26.9 ± 3.7
K_e_	h^-1^	1.8 ± 0.4	0.9 ± 0.1	1.3 ± 0.2
Vd	L/Kg	-	-	2.0 ± 0.2
Cl	mL/min	-	-	13 ± 3
t_1/2 a_	min	-	51 ± 17	-
t_1/2 e_	min	27 ± 11	38 ± 3	33 ± 5
MRT	min	49 ± 18	69 ± 5	57 ± 11
AUC_tot_	μg min^-1^/mL	157 ± 70	748 ± 84	1245 ± 206
F% orally	%	13%	-	-

Subsequently, the software Kinetica 5.0 was used to establish the pharmacokinetic model of ***C2***, which showed that the plasma concentration declined bi-exponentially. Specifically, the parameters α = 0.037 and β = 0.011 min^-1^ were determined, and the values K_12_ = 0.008 min-1 and K_21_ = 0.022 min^-1^ were calculated; the elimination half-life α was t_1/2α_ = 18 min, and the elimination half-life β was t_1/2β_ = 62 min

#### Tissue Distribution

**Fig 4:**
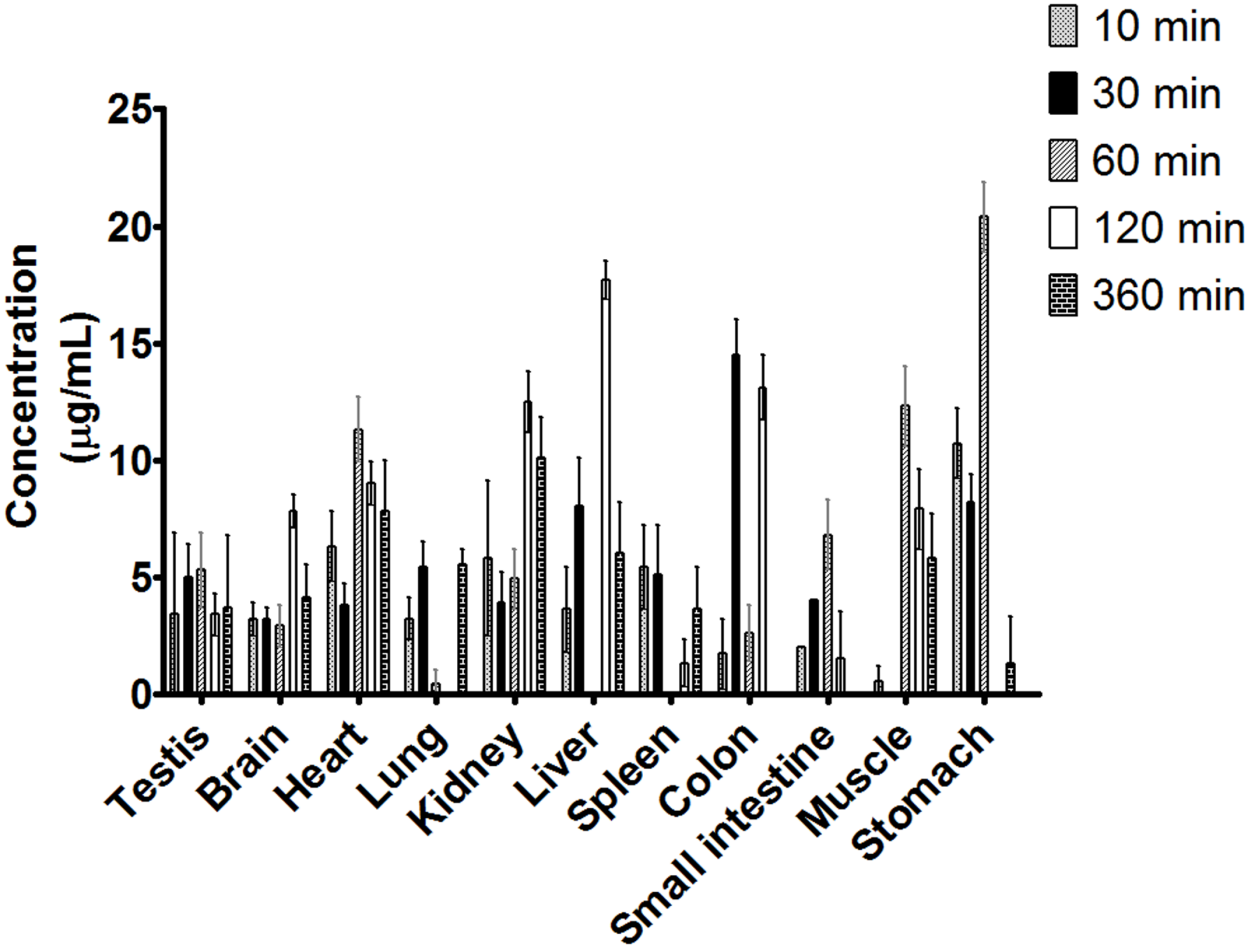
Concentration of *C2* in different rat tissues 10, 30, 60, 120 and 300 min after receiving a single i.g. dose of the compound at 100 mg/kg. (Mean ± SD, n = 3).

**Fig 5:**
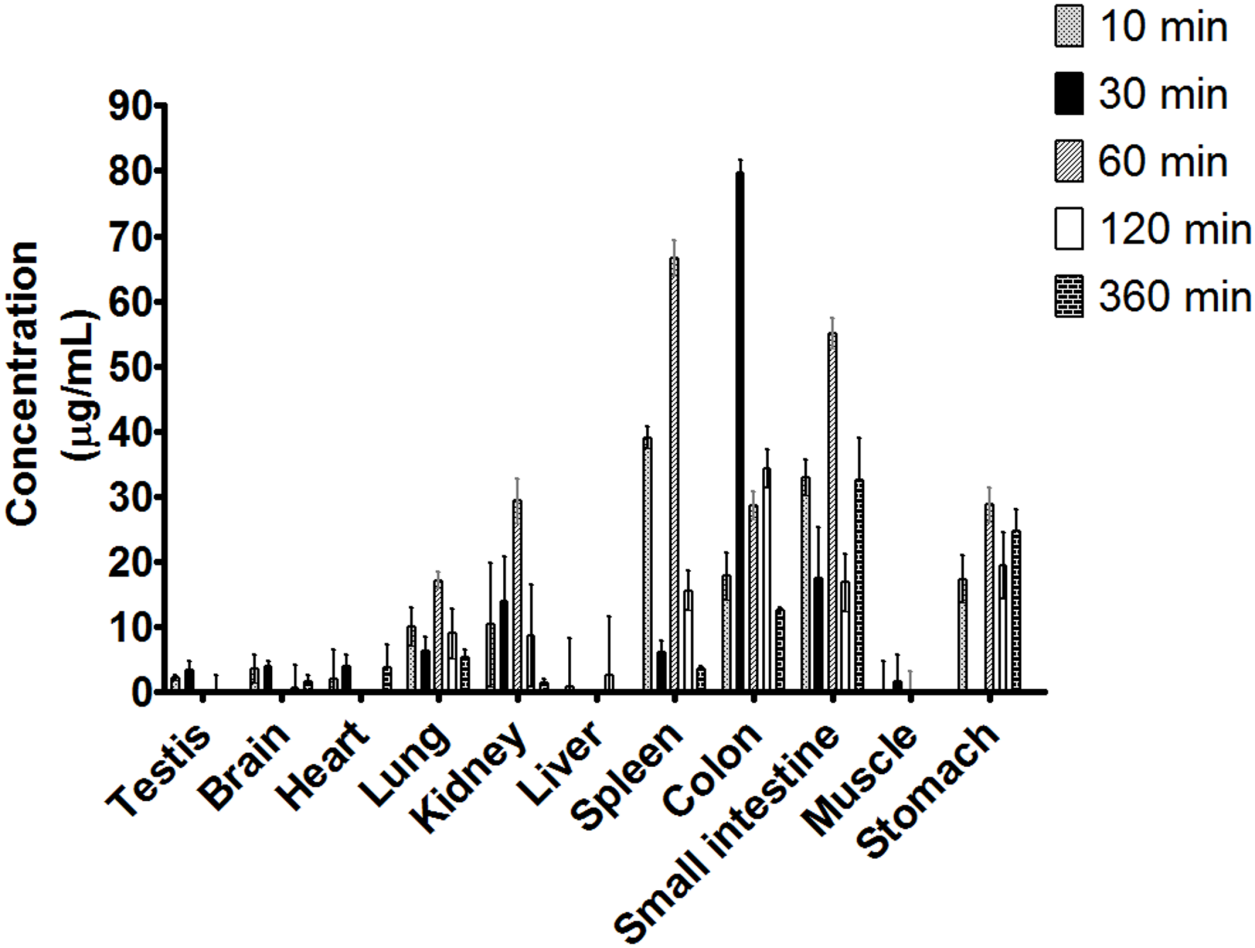
Concentration of *C2* in the different rat tissues obtained at 10, 30, 60, 120 and 300 min after receiving a single i.p. dose of the compound at 100 mg/kg. (Mean ± SD, n = 3).

This study assessed two conditions, i.g. (Fig 4) and i.p. (Fig 5), at different time points (10, 30, 60, 120 and 360 min). Before the 2 h time point, ***C2*** was widely distributed in all examined tissues for both administration routes (i.g., and i.p.), and the maximum concentration decreased throughout the 6 h of monitoring. The results show the presence of ***C2*** in the brain and testicles, indicating that ***C2*** effectively crossed the blood-brain barrier and hemato-testicular barrier. For routes of administration, ***C2*** tended to accumulate in almost all tissues examined. After i.g. administration, a higher concentration of ***C2*** was detected in the liver, and a similar concentration of ***C2*** was detected in the stomach; moreover, i.g. administration resulted in lower ***C2*** concentrations in the colon and small intestine than i.p. administration, suggesting that the stomach is the absorption site of ***C2***.

#### Protein binding and Blood-Plasma partitioning (BP ratio)

The protein-binding data of ***C2*** are summarized in Table 5. The unbound fraction of ***C2*** at concentrations of 1 to 20 μg/mL ranged from 89.8% to 92.5%, meaning that this fraction of ***C2*** is available to cross tissues.

**Table 5:** Bound and unbound fractions of *C2* in rat plasma and its blood-plasma partition ratio.

Concentration μg/ mL	% Unbound fraction of *C2* in rat plasma ± SD	% Bound fraction of *C2* in rat plasma ± SD
**1**	92.1 ± 2.3	7.9 ± 2.3
**5**	92.5 ± 1.8	7.5 ± 1.8
**10**	91.9 ± 0.7	8.1 ± 0.7
**20**	89.8 ± 0.8	10.2± 0.8

The blood-plasma partitioning (BP ratio) of ***C2*** in rats was experimentally measured and ranged from 0.6 to 0.71 (Table 6). A BP ratio less than 1 indicates that the compound is free in the plasmatic phase and not inside the blood cells [30].

**Table 6:** Blood-plasma partitioning (BP ratio) of *C2* in Wistar rats.

Concentration (μg/mL)	BP ratio ± SD
5	0.71 ± 0.01
10	0.60 ± 0.03

## Discussion

The pharmacological effect of a drug has been described to depend on its pharmacokinetic properties, such as good oral absorption, an appropriate half-life of elimination, good distribution and good bioavailability. The compound ***C2*** (5-[(4-carboxybutanoyl)amino]-2-hydroxybenzoic acid) is a novel synthetic derivative of 5-aminosalicylic acid (5-ASA) that has shown *in vitro* and *ex vivo* dual activity as an antioxidant and MPO inhibitor [34–36]. To continue the preclinical study of this compound, we herein reported its pharmacokinetic study in Wistar rats.

For ***C2*** quantification in plasma and other tissues, a RP-HPLC method with UV-Vis detection was first developed, and the validation demonstrated that this method is suitable and complies with the performance parameters established by the FDA and ICH regulations for bioanalytical methods.

In this study, three routes of administration were examined, intragastric, intravenous and intraperitoneal, to delineate the absorption process in the gastrointestinal tract and intraperitoneal tissues. When ***C2*** was administered via the intragastric route, the compound quickly reached the systemic circulation, but unfortunately, the oral bioavailability was only 13% of the administered dose; however, ***C2*** was well distributed in all tissues and able to cross the blood brain barrier. More importantly, the compound reached the small intestine and colon. Therefore, the use of this compound is advantageous to that of 5-ASA for the treatment of UC and CD. Specifically, ***C2*** presented a t_1/2_ of approximately 33 ± 5 min, and the Vd was 2.0 L/kg. ***C2*** has a short half-life, which may be a disadvantage with respect to 5-ASA for setting the dosing regimen. However, the plasma half-life of salicylates, such as aspirin, is 15 min, and that of salicylate 2–3 h at low doses. Furthermore, ***C2*** was no longer detected in the plasma 6 h after administration, indicating that ***C2*** accumulates in some tissues [44]. This feature may be a disadvantage for toxic compounds, but the acute toxicity study demonstrated that ***C2*** has a low acute toxicity risk. Therefore, the tissue accumulation of ***C2*** may be an advantage, especially for establishing the dosing regimen.

We hypothesize that a fraction of the administered ***C2*** dose is absorbed in the stomach, whereas the remaining fraction reaches the small intestine and colon, where it accumulates for with 6 h or longer. In addition, we associate the low intragastric absorption of ***C2*** with the difficulty to dissolve this compound in isotonic saline solution; therefore, a formulation based on 75% saline (0.9%), 5% Tween 80 and 20% propylene glycol was used according to the proportions permitted for administration in humans [45]. In addition the pH may affect the bioavailability of ***C2*** because this compound is entirely unionized at pH > 3. With respect to intraperitoneal administration, ***C2*** was absorbed at neutral pH despite the fact that it was mostly ionized. However, the hydroxyl group that is attached to the aromatic ring is not ionized, which may promote the absorption of this molecule.

The protein-binding study showed a lower ***C2*** protein binding of 10% than that of 5-ASA (40%), indicating that more ***C2*** would be available to cross tissues and reach the site of action. This phenomenon may arise because drugs are generally weak acids, which bind almost exclusively to the albumin plasma protein that is readily bound to acidic drugs. The binding of drugs to albumin is generally reversible and favored by lipid solubility [46, 47]. Therefore, compounds with low lipo-solubility, such as ***C2*** (Log P = 1.3), are less likely to bind to albumin. Finally, a BP-ration less than 1 indicates that the compounds are free in the plasmatic phase and not inside blood cells, which favors ***C2*** reaching the site of action in a live organism, including humans [42, 43].

## Conclusions

In the present study, a bioanalytical method to quantitate ***C2*** (5-[(4-carboxybutanoyl)amino]-2-hydroxybenzoic acid) in rat plasma with an HPLC technique was validated to meet the specified performance parameters for bioanalytical applications provided for the pharmacokinetic evaluation of this new chemical entity. Pharmacokinetic studies showed that the compound is absorbed by the biological membranes and widely distributed in all tissues and that its binding to albumin was minimal.

In summary, we found several important differences and advantages of ***C2*** compared with 5-ASA based on its pharmacokinetic properties in Wistar rats. Considering the pharmacological activity and acute toxicity studies, we propose the use of ***C2*** as a potential candidate to treat inflammatory diseases, including UC and CD.

## Supporting Information

S1 File Committee_CICUAL_opinion. (PDF)

## Abbreviations

4-ABAH: 4-aminobenzoic acid
AD: Crohn's disease
5-ASA: aminosalicylic acid
AUC_0-t_: area under the plasma concentration-time curve from time zero to the last measurable concentration
AUC_tot_: the area under the plasma concentration-time curve from time zero to infinity
BP ratio: blood-plasma partitioning
***C2***: 5-[(4-carboxybutanoyl)amino]-2-hydroxybenzoic acid
CL: clearance
C_max_: maximum plasma concentration
HOCl: hypochlorous acid
MRT: mean residence time
MPO: mieloperoxidase
HPLC: high performance liquid chromatography
RP-HPLC: reverse-phase high resolution liquid chromatography
SP: sulfapyridine
t_1/2_: elimination half-life
T_max_: time to reach C_max_
UC: ulcerative colitis
Vd: apparent volume of distribution
