# Supplementary material for: Pharmacokinetics in Wistar Rats of 5-[(4-Carboxybutanoyl)Amino]-2-Hydroxybenzoic Acid: A Novel Synthetic Derivative of 5-Aminosalicylic Acid (5-ASA) with Possible Anti-Inflammatory Activity
Source: PLoS One. 2016 Jul 25;11(7):e0159889. doi: 10.1371/journal.pone.0159889 (PMC4959752; doi:10.1371/journal.pone.0159889)
Supplement: S1 File — (PDF) [file pone.0159889.s001.pdf]

México, D.F., a 27 de julio de 2015.

Dictamen: CICUAL-01/27-07-2015.

El Comité Interno para el Cuidado y Uso de Animales de Laboratorio (CICUAL) de la Escuela Superior de Medicina del Instituto Politécnico Nacional constituido por: Dr. Carlos Vera Arzave, Dr. Ángel Iván Orlando Rubio Gayosso, Dr. Pedro López Sánchez , Dr. Joel Lomelí González, y el Dr. Gustavo Guevara Balcázar, en su sesión celebrada el día 27 de julio del presente año, declara haber evaluado el protocolo No. ESM.CICUAL-01/27-07-2015 del proyecto: **"Evaluación *ex vivo* y estudio farmacocinético de dos derivados del ácido 5-aminosalicílico considerados como potenciales agentes terapéuticos"**. Presentado por la Investigadora. ***Dra. Jessica Elena Mendieta Wejebe.***

Para su evaluación la comisión del CICUAL revisó los siguientes antecedentes: lógica con el uso de sustancias, inoculaciones, alojamiento y cuidado, número de animales, especie, cepa y procedencia y punto final humanitario. Después de dicho análisis se emitió el siguiente dictamen:

ACEPTADO

1.- Protocolo No. ESM-CICUAL-01/23-06-2015. Versión 1.0, con fecha de junio del 2015.
2.- Protocolo No. ESM-CICUAL-01/27-07-2015. Versión 2.0, con fecha de julio del 2015.

Atentamente

INSTITUTO POLITECNICO NACIONAL
ESTADOS UNIDOS MEXICANOS
SEP
ESCUELA SUPERIOR DE MEDICINA
COMITE INTERNO PARA EL CUIDADO
Y USO DE ANIMALES DE LABORATORIO

D. en C. Carlos Vera Arzave
Presidente del Comité

CVA/jod.
